# CONTENT AND OUTCOME OF NON-PHARMACOLOGICAL REHABILITATION FOR WOMEN WITH TRAUMATIC BRAIN INJURY – A SCOPING REVIEW

**DOI:** 10.2340/jrm.v58.45767

**Published:** 2026-10-02

**Authors:** Kajsa LIDSTRÖM HOLMQVIST, Marie MATÉRNE, Grahame SIMPSON, Jessica MASSEY, Thomas STRANDBERG

**Affiliations:** 1 University Health Care Research Centre and Department of Neurology and Rehabilitation Medicine, Faculty of Medicine and Health, Örebro University, Örebro; 2 School of Behavioural, Social and Legal Sciences, Örebro University, Örebro, Sweden; 3 Liverpool Brain Injury Rehabilitation Unit, South Western Sydney Local Health District, Sydney; 4 John Walsh Centre for Rehabilitation Research, School of Health Sciences, Faculty of Medicine and Health, The University of Sydney, Sydney; 5 Brain Injury Rehabilitation Research Group, Ingham Institute for Applied Medical Research, Sydney, Australia; 6 Disability Research, Örebro University, Örebro, Sweden

**Keywords:** brain injuries, female, gender, intervention

## Abstract

**Objective:**

To identify and map evidence on the content and outcomes of non-pharmacological rehabilitation for women with traumatic brain injury.

**Design:**

Scoping review.

**Subjects/patients:**

Women aged 18–65 years with traumatic brain injury of any severity.

**Methods:**

The PubMed, CINAHL and PsycINFO databases were searched. Studies including individuals with nontraumatic brain injuries, text and opinion papers, conference abstracts, study protocols, and grey literature were excluded. Inclusion criteria were: either ≥ 50% of the sample were women, or outcome data distinguished women’s results from the rest of the sample. A data-extraction form was used. Extracted data included specific details concerning the participants, context, study methods, and key findings.

**Results:**

41 studies with varying designs were included. None of the interventions described had a specific focus on women or women’s needs. Community or outpatient settings dominated. The interventions were grouped into 3 main categories with underlying sub-categories – *Alternative and complementary interventions*, *Training to restore functions*, and *Skills training to enhance cognitive functioning* – with the latter category containing the most studies.

**Conclusion:**

The interventions described targeted a wide range of consequences from traumatic brain injury but without a clear gender perspective. Current rehabilitation models need to be developed to also include such a perspective.

## LAY ABSTRACT

This article reviews the literature on rehabilitation interventions for women aged 18–65 years who have sustained a traumatic brain injury. Three databases were searched to find studies describing interventions. Only studies in which at least half the participants were women or the results for women were separated from those for men were included. Forty-one studies are described in the results, categorized based on target area: *Alternative and complementary interventions*, *Training to restore functions*, and *Skills training to enhance cognitive functioning*. The third category contained the most studies. The results show that none of the studies were specifically developed for or adapted to women’s needs. As women experience somewhat different symptoms and consequences in life from traumatic brain injury in comparison with men, this study emphasizes the clinical and research importance of developing and evaluating interventions with a gender perspective.

Traumatic brain injury (TBI) is a leading cause of long-term disability and mortality worldwide and has been identified as a significant global health concern (1). The severity of TBI can be classified as mild, moderate, or severe, with severe TBI indicating significant consequences in terms of long-term effects on motor, sensory, cognitive, communication, behavioural, and social factors. TBI rehabilitation is recommended to be delivered by a multidisciplinary team with a focus on functional recovery and psychosocial adjustment guided by person-centred goals (2–5). Approaches include attempts to restore functions (e.g., motor skills; coordination, and balance) or to train individuals to learn to use compensatory techniques such as assistive technology or metacognitive strategies (6). Psychosocial adjustment encompasses the process of adapting to changes in identity, emotional well-being, and social roles following injury, with the aim of promoting participation in daily life activities and quality of life (7).

In TBI rehabilitation, the influence of both sex and gender has been underappreciated where gender extends beyond biological sex and integrates the social, biological, cultural, and behavioural factors that shape rehabilitation processes and outcomes (8). There are important reasons to apply a gendered perspective to neurorehabilitation in the context of current disparities between females and males across impairments, functional/psychosocial outcomes, and rehabilitation treatments (9, 10). The need to develop intervention strategies to address these disparities for women after acquired brain injury (ABI) has been identified as a global health issue across neurorehabilitation, as identified by both the World Stroke Organization (11) and the Lancet Neurology Commission in Traumatic Brain Injury (TBI) (12). In relation to TBI, there may be multiple factors that contribute to these disparities. For instance, sex-specific biological and social factors can affect both TBI rate and recovery (13). For example, hormonal disturbances due to TBI in women have been suggested as an explanation for some differences in sex-related outcomes. Moving on from possible biological factors, another difference lies in the epidemiology of TBI, with population-based prevalence estimates showing that men are more than twice as likely to be affected (15–17). This may be another contributing factor to the paucity of studies conducted on women with TBI, which has led to an incomplete understanding of the impact of TBI in females (13, 14).

In addition to the variations in incidence rates, gender disparities in the causes of TBI have also been identified, with women being more likely than men to sustain TBI due to intimate partner violence (18, 19). Furthermore, symptom profiles can also vary by gender, with women reporting higher levels of anxiety, depression, and post-concussion symptoms after TBI. Women have also reported poorer outcomes than men even though the size of the differences differs depending on age and severity of injury with women of child-bearing age with mild TBI showing poorer outcomes (20). Women also report high degrees of social isolation, loss of friendships, and difficulties in adjusting to a new sense of self while feeling not understood or trivialized (14).

Given this context of gender-based disparities, and the call for intervention strategies to address such disparities, this scoping review aimed to identify and map evidence on the content and outcomes of non-pharmacological rehabilitation for women under 65 years of age with TBI, guided by the following research questions:

- What is the content of rehabilitation delivered to women with TBI?
- What are the outcomes of rehabilitation for women with TBI?
- What experiences of rehabilitation content, outcome, and support are reported by women with TBI?

## METHODS

This scoping review follows the methodological guidelines of the Joanna Briggs Institute (JBI) (21, 22) and is reported in accordance with the Preferred Reporting Items for Systematic Reviews and Meta-Analyses – Extension for Scoping Reviews (PRISMA-ScR) guidelines (23). The analytical process was supported by the Covidence software. The study is registered in Open Science Direct (https://doi.org/10.17605/OSF.IO/QUY3T), and a study protocol has been published (24).

Eligibility criteria were structured by participants, concept, and context, according to the recommendations for scoping reviews by Peters et al. (22).

### Eligibility criteria: participants

This scoping review included studies in which at least half of the sample were adult women (aged 18–65 years) who had sustained a TBI of any severity, or in which outcome data were reported by gender, distinguishing women’s results from the rest of the sample. Studies with a mixed sample including individuals with non-TBI were excluded.

### Eligibility criteria: concept

*Content of non-pharmacological rehabilitation interventions.* This review considered studies that described the content of all types of non-pharmacological rehabilitation interventions delivered to women, except invasive treatments such as brain stimulation or acupuncture, treatment at emergency units, or interventions targeting secondary diagnoses such as depression.

*Outcome of rehabilitation interventions.* Relevant outcomes covered both quantitative and qualitative measures, including scores on measures of function or participation, quality of life, coping strategies or cognitive strategies, as well as key themes elicited from qualitative research.

### Eligibility criteria: context

Studies in which participants received a rehabilitation intervention in hospital (inpatient and outpatient) or in community-based settings were considered. No geographical restriction was placed on included studies.

### Types of sources

Peer-reviewed experimental and quasi-experimental study designs – including randomized controlled trials (RCTs), non-randomized controlled trials, pre–post studies, and interrupted time-series studies – were included, as well as cohort studies, case control studies, cross-sectional studies, and case reports. Qualitative studies with various designs were also included. Text and opinion papers, conference abstracts, grey literature, and study protocols were excluded.

### Search strategy

Only studies in English were included, as most research is published in English. Studies published from the year 2000 onwards were included, as studies began more frequently to apply a gender perspective at the turn of the millennium.

The PubMed (PubMed), CINAHL (EBSCO), and PsycINFO (EBSCO) databases were searched using a search strategy developed in collaboration with research librarians, who also conducted all searches. An initial limited pilot search of PubMed was undertaken to ensure that relevant articles were included. The text words contained in the titles and abstracts of relevant articles, as well as the index terms describing the articles, were used to develop a full search strategy for the 3 databases. This search strategy, including all identified keywords and index terms, was applied for each included database. The main search was conducted in February 2024, and an updated search was completed in October 2025. Searches for each database are presented in Appendix S1.

### Study selection

All identified studies were collated and uploaded into Covidence (25), and duplicates were removed. Three independent reviewers (KLH, MM, TS) assessed the titles and abstracts against the eligibility criteria. One reviewer (KLH) screened all titles and abstracts, while the other 2 reviewers (MM, TS) screened half each. To determine whether most participants in a study were women/female and/or whether the study outcomes were reported separately by sex/gender, it was sometimes necessary to screen the methods and result sections as well. A few disagreements were resolved through joint discussion. Potentially relevant studies were read in full and assessed against the eligibility criteria using the same procedure as in the previous step. In studies selected for data extraction, manual searches of reference lists were conducted to identify additional studies of interest.

### Data extraction

Data were extracted by 2 reviewers (KLH, TS) using a data-extraction form (24) adapted by the reviewers from Peters et al. (22). Extracted data included specific details regarding the participants, context, study methods, and key findings relevant to the review questions. The data-extraction method was piloted on 10 articles before further use, and no further modifications of the extraction form were made. The 2 reviewers compared their data extractions and agreed on a joint extraction.

### Data analysis and presentation

Information on authors, year, country of origin, setting, aim, type of studies, methods, population/severity of TBI, intervention descriptions, outcome measures, and quantitative and qualitative outcomes are presented in Tables I and II. The content and outcome of each intervention were analysed and categorized according to intervention type. Many interventions included more than 1 type (e.g., both restorative and skills-directed content) and were categorized based on their primary target area or content. The authors jointly determined the primary focus of each study. Two authors (KLH, TS) performed an initial categorization, which was discussed and refined by all authors until consensus was reached. For case studies, only results applying to female participants are reported here. Studies on participants’ experiences of the rehabilitation content and outcome were very few and were summarized on a manifest level. In addition, such studies reported on experiences from interventions that were evaluated with quantitative outcomes in other included studies. The results from each intervention are therefore reported together, including both participants’ experiences and quantitative outcomes.

**Table I:** Studies included in this review, in alphabetical order

Reference	Country	Context	Aim	Severity of TBI	Sample size, gender distribution^a^, age^b^	Design
Alarie et al., 2022 (54)	Canada	Outpatient	To determine the feasibility, safety, and acceptability of a remotely delivered progressive walking intervention designed for adults with persistent mTBI symptoms and explore its effects on health-related outcomes	Mild	*N* = 20 16 F (female), 80% Mean age 42.5	Feasibility study
Banerjee et al., 2021 (26)	India	Clinic	To study multiple aspects of individuals’ functioning along with cognitive functioning to help restore the “sense of self”, while carrying out neuropsychological rehabilitation	Mild to moderate	*N* = 1 1 F, 100% Age 25	Case study
Bay, et al., 2016 (40)	USA	Outpatient	To describe 2 lifestyle interventions, their methods of delivery and participants’ levels of satisfaction for persons with chronic TBI	Mild to moderate	*N* = 25 12, F, 68% Age 18–80	RCT
Bédard et al., 2003 (50)	Canada	Community	To examine the potential efficacy of a mindfulness-based stress-reduction approach to improve quality of life in individuals who have suffered TBI	Mild to moderate	*N* = 10 7 F, 70% Mean age 43	Pre–post study
Bieman-Copland & Dywan, 2000 (27)	Canada	Community	To describe the implementation of a no-confrontative behavioural approach to treat anosognosia	Severe	*N* = 1 1 F, 100% Age 28	Case study
Bonn et al., 2023 (51)	Canada	Outpatient	To determine the number and types of goals by individuals completing an interdisciplinary outpatient rehabilitation intervention for persisting symptoms following mTBI	Mild	*N* = 217 154 F, 71% Mean age 44.4	Pre–post study
Bonn et al., 2025 (53)	Canada	Outpatient	To evaluate whether factors associated with developing persistent symptoms influence satisfaction with the performance of self-identified goals	Mild	*N* = 198 144 F, 73% Mean age 44.7	Pre–post study
Briggs et al., 2024 (63)	USA	Outpatient	To demonstrate changes in vision efficiency and patient reports of vision-related occupational performance in adult patients with an mTBI after occupational-therapy-led Remedial Vision Rehabilitation	Mild	*N* = 11 9 F, 82% Mean age 54	Pre–post study
Brown et al., 2015 (41)	USA	Community	To test whether a curriculum-based advocacy training programme improves advocacy behaviour when compared with a matched group engaged in self-directed advocacy activities	Moderate to severe	*N* = 129 78 F, 60% Age >18	RCT
Callahan et al., 2023 (42)	USA	Community	To investigate the LoveYourBrain Mindset programme’s feasibility, acceptability, usability, and effectiveness in improving TBI clinical outcomes	Any severity	*N* = 411 335 F, 82% Mean age 45	Pre–post study
Campbell et al., 2022 (39)	USA	Clinic	To explore feasibility and adoption of an in-person rehabilitation programme for subacute mTBI delivered through tele-rehabilitation	Mild	*N* = 56 (in person) 47 F, 84% Mean age 34 *N* = 17 (tele-rehab.) 9 F, 53% Mean age 38	Feasibility study
Cantor et al., 2014 (43)	USA	Community	To determine whether the Short-Term Executive Plus cognitive rehabilitation programme improves executive dysfunction after TBI	Any severity	*N* = 98 61 F, 62% Mean age 45	RCT
Connolly & Lyon, 2017 (28)	USA	Outpatient	To describe a patient with significant visual symptoms caused by accommodative and vergence dysfunction that was treated by vision therapy post-mTBI	Mild	*N* = 1 1 F, 100% Age 20	Case study
Dawson et al., 2009 (52)	Canada	Community	To test the applicability of the Cognitive Orientation to Occupational Performance approach for use with adults with executive dysfunction arising from TBI	Mild	*N* = 3 1 F, 33% Age 32	Case study
Donnelly et al., 2021 (44)	USA	Community	To conduct a mixed-method, pre–post, retrospective study on the feasibility, acceptability, and effectiveness of the LoveYourBrain yoga programme	Any severity	*N* = 705 529 F, 75% Age 15–70	Mixed method
Esquenazi et al., 2013 (45)	USA	Outpatient	To compare the effects of robotic-assisted treadmill training (RATT) and manually assisted treadmill training (MATT) in participants with TBI and to determine the potential impact on the symmetry of temporal walking parameters	Not specified	*N* = 8 (RATT) 5 F, 62,5% Mean age 37 *N* = 8 (MATT) 4 F, 50% Mean age 42	RCT
Faulkner et al., 2025 (60)	New Zealand	Outpatient	To achieve focus of treatment by embedding an Acceptance and Commitment Therapy, ACT-informed intervention for mTBI into multidisciplinary outpatient rehabilitation services	Mild	*N* = 24 15 F, 62.5% Mean age 32	Feasibility study
Faulkner et al., 2025 (61)	New Zealand	Outpatient	To understand participants’ experiences of ACTion mTBI, (Acceptance and Commitment Therapy for people with Mild Traumatic Brain Injury), determine acceptability, and identify any refinements needed to inform a full-scale effectiveness trial	Mild	*N* = 23 21 F, 91% Mean age 34	Qualitative study
Fleming et al., 2005 (56)	Australia	Outpatient	To describe a prospective memory rehabilitation programme which incorporates elements of self-awareness and compensatory strategy training	Not specified	*N* = 3 1 F, 33% Age 52	Pre–post study
Finch et al., 2017 (55)	Australia	Outpatient	To evaluate whether a novel meta-cognitive, goal-based intervention improved and maintained the social communication skills of adults with TBI	Not specified	*N* = 8 4 F, 50% Age 35–49	Pilot study

Fritz & Basso, 2013 (29)	USA	Clinic	To determine the feasibility and potential value of incorporating a cognitive-motor dual-task training programme into physical therapy for a patient with a severe TBI	Severe	*N* = 1 1 F, 100% Age 25	Case study

Grimm et al., 2017 (36)	USA	Not specified	To analyse changes in emotional regulation and health-related quality of life following a yoga intervention for participants with TBI	Not specified	*N* = 3 2 F, 66% Mean age 44	Mixed method
Hecker et al., 2025 (65)	The Netherlands	Outpatient	To investigate the effectiveness and feasibility of individual 14-week exposure therapy for patients with persistent symptoms after concussion	Mild	*N* = 4 3 F, 75% Mean age 63	Single-case experimental design
Imhoff et al., 2017 (30)	Canada	Outpatient	To evaluate the effectiveness of an in-simulator training programme with automated feedback on driving performance in a TBI individual	Moderate	*N* = 1 1 F, 100% Age 20	Case study
Kersey et al., 2025 (64)	USA	Community	To modify the intervention ENGAGE for the TBI population and evaluate its feasibility. ENGAGE-TBI, a community-based group intervention for people with traumatic brain injury	Not specified	*N* = 12, 6 F, 50% Mean age 47	Feasibility study
King et al., 2024 (66)	The Netherlands	University faculty	To investigate the efficacy of a newly developed intensive exposure intervention for individuals with persistent concussion symptoms after concussion, aiming to tackle avoidance behaviour	Mild	*N* = 4 3 F, 75% Mean age 26.5	Single case experimental design
Kreutzer et al., 2018 (46)	USA	Outpatient	To evaluate the efficacy of a structured, curriculum-based intervention to promote post-injury resilience and adjustment	Any severity	*N* = 83 46 F, 55% Mean age 42	RCT
Mahajan et al., 2024 (31)	India	Outpatient	To investigate the effectiveness of an integrated psychotherapeutic approach combining Rational Emotive Behavior Therapy and Cognitive Retraining in managing the complex psychological effects of TBI	Not specified	*N* = 1 1 F, 100% Age 34	Case study
Nguyen et al., 2024 (57)	Australia	Community	To explore participant experiences of Interdisciplinary Rehabilitation for Concussion Recovery, an interdisciplinary intervention for post-concussion symptoms and its impact on symptoms, daily function and concussion beliefs	Mild	*N* = 13 8 F, 61% Mean age 39.7	Qualitative study
Ownsworth et al., 2010 (58)	Australia	Community	To evaluate the effects of meta-cognitive skills training and behavioural practice on error self-regulation during a naturalistic task after TBI	Severe	*N* = 3 1 F, 33% Age 43	Case study
Palmese & Raskin, 2000 (35)	USA	Community	To investigate the success of Attention Process Training in the rehabilitation of attention in individuals diagnosed with mTBI	Mild	*N* = 3 1 F, 100% Age 42	Case study
Raina et al., 2016 (47)	USA	Community	To evaluate the feasibility of conducting a randomized clinical trial of an internet-based manualized intervention to teach individuals with TBI to manage fatigue	Any severity	*N* = 17 (Maximising Energy) 9 F, 53% Mean age 44	RCT
Rath et al., 2003 (34)	USA	Outpatient	To evaluate the efficacy of a remedial programme aimed at reducing difficulties in emotional self-regulation and facilitating problem-solving, compared with a conventional neuropsychological rehabilitation programme	Mild	*N* = 60 37 F, 62% Mean age 43.6	RCT
Sartor-Glittenberg & Brickne, 2014 (48)	USA	Outpatient	To describe changes in impairments and activity limitations in individuals with severe cerebellar ataxia from TBI who participated in a long-term, multidimensional physical therapy programme	Severe	*N* = 3 1 F, 100% Age 22	Case study
Schmid et al. 2016 (33)	USA	Community	To investigate the feasibility of people with TBI completing an 8-week yoga programme and to explore change in physical functioning after the programme for people with chronic TBI	Not specified	*N* = 3 2 F, 75% Age 42, 48	Mixed method
Tate et al. 2020 (59)	Australia	Community	To develop a goal-directed intervention to increase non-vocational activity for people with TBI who experienced substantial disability and evaluate its effectiveness.	Severe	*N* = 7 1 F, 14% Age 55	Single-case experimental design
Toglia et al. 2010 (38)	USA	Community	To operationalize and explore the multicontext approach to generalization of trained strategies among individuals with TBI	Moderate	*N* = 4 2 F, 50% Age 29, 50	Pre–post study
Toglia et al. 2011 (49)	USA	Community	To examine whether an individual could learn to use a checklist across variable functional situations, perform better in functional tasks, and report increased levels of self-regulation and everyday functioning	Moderate	*N* = 1 1 F, 100% Age 29	Case study
Tsaousides et al. 2017 (37)	USA	Community	To perform a preliminary evaluation of the efficacy of a web-based group intervention to improve emotion regulation in individuals with TBI	Any severity	*N* = 91 51 F, 56% Mean age 47	Pre–post study
Tzeng et al. 2025 (62)	Taiwan	Outpatient	To evaluate the effects of a 12-week digital walking exercise intervention incorporating real-time self-monitoring and personalized feedback via wearable fitness trackers compared with standard exercise education	Mild	*N* = 52 30 F, 57,7% Mean age 43.98	RCT
Özata Değerli et al. 2025 (32)	Türkiye	Outpatient	To examine the effect of multicontext occupational therapy intervention on cognitive function, occupational performance, and independence in activities of daily living in a client with TBI	Not specified	*N* = 1 1 F, 100% Age 42	Case study

a Sample size and gender distribution is reported as presented in the different studies, but if different groups (e.g., control group) are presented, the target group for this study only is presented in the table.

b Reporting of age varies in the different studies and is reported in this table as presented in the original studies.

**Table II:** Interventions per category

Reference	Design	Intervention description, length of intervention	Outcome measures	Outcomes
Alternative and complementary interventions (*n* = 6)				
Yoga (*n* = 4)				
Callahan et al., 2023 (42)	Pre–post study	On-line manualized group-based, yoga, mindfulness, and psychoeducation programme to people with TBI, caregivers, and clinicians. 6 weeks	Acceptability: single question Quality of Life after Brain Injury (QOLIBRI) Quality of Life in Neurological Disorders (Neuro-QOL) TBI-QOL(Quality of Life)	Acceptability 9 out of 10. Statistically significant improvement in quality of life, resilience, emotional and behavioural dysregulation, cognition, and positive affect and well-being. Interactive classes with group discussions showed better quality of life, no other differences due to choice of interactive class
Donnely, et al., 2021 (44)	Mixed method	Each class with a specific structure of breathing exercises, gentle yoga, guided meditation, and facilitated discussion with psychoeducation to cultivate skills in resilience. 6 weeks once a week.	Quality of Life after Brain Injury – Overall Scale (QOLIBRI-OS) Neuro-QOL TBI-QOL	Statistically significant improvements in QOLIBRI-OS, resilience, positive affect and well-being, and cognition. Better ability to regulate anxiety, anger, stress, and impulsivity. Improved memory, concentration, and self-awareness. Greater motivation to participate in activities in the community
Grimm et al., 2017 (36)	Mixed method	Protocol-based individual yoga tailored to the individual’s needs and abilities. All sessions included breathing, yoga postures and meditation/relaxation. 8 weeks, 4 sessions, 1 h bi-weekly.	Difficulties in Emotion Regulation Scale (DERS) QOLIBRI Semi-structured interview	Emotional regulation: inconsistent results. Quality of life: 6%–8% improvement Interviews: positive experiences of improved peacefulness, personal growth, mind–body connection, emotional regulation, concentration, self-confidence, and social participation
Schmid et al., 2016 (33)	Mixed method	Protocol-based individual yoga tailored to the individual’s needs and abilities. All sessions included breathing, yoga postures, and meditation/relaxation. 8 weeks, 16 sessions 1 h twice/week	Berg Balance Scale (BBS) Activities-specific Balance Confidence Scale (ABC) Pain, Enjoyment of Life and General Activity Scale (PEG) Range of Motion (ROM) Goniometer Chair to stand test Gait speed test Interviews	Improved balance. Inconsistent pain results. Increased flexibility, strength, and gait speed. Experiences of positive impact on participants’ life and recovery, increased strength, less fear of falling, better walking ability, better activity performance
Mindfulness (*n* = 2)				
Bay et al., 2016 (40)	RCT	Mindfulness Group Therapy: Introduction to bodily sensations and mindfulness. Positive body awareness and movement. Single focused meditation using a mantra and self-compassionate meditation on positive affirmations. 8 weeks, 90 min/week, after 4 weeks, 60 min/week	Center for Epidemiological Studies – Depression Scale (CES-D) Rivermead Post-Concussion Questionnaire (RPQ) Study-specific survey on satisfaction	Decreased depression and post-concussion symptoms. No change in psychological functioning. Overall satisfaction with the intervention
Bédard et al., 2003 (50)	Pre–post study	Manual-based intervention following Kabat-Zinn’s Mindfulness-based Stress Reduction Programme and Kolb’s Experiential Learning Cycle. Insight meditation, breathing exercises, guided visualization, and group discussion as the primary techniques. 12-weekly group sessions	Short-form Health Survey (SF-36) Beck Depression Inventory (BDI-II) Symptom Check List – 90 items (SCL-90-R) Perceived Stress Scale (PSS) Multidimensional Health Locus of Control Scale (MHLC) Global Severity Index (GSI) Positive Symptom Distress Index (PSDI) Community Integration Questionnaire (CIQ)	Improved quality of life and cognitive affective domain of BDI-II. No improvements in overall BDI-II, PSS, GSI scores. Improved PSDI. No changes in CIQ. No changes between groups in MHLC
Training to restore functions (*n* = 13)				
Training to restore physical functions (*n* = 11)				
Alarie et al. 2022 (54)	Feasibility study	Intervention was remotely delivered and consisted of weekly telehealth sessions with participants to individualize their walking progression and offer motivational support specific to individuals with an mTBI. 8 weeks	Walking log. Number of minor falls. Perceived exacerbation of symptoms. Acceptability of the intervention. 17-item 5-point scale designed for this study	In total, 38% of participants completed the intervention and found it to be feasible, safe, and acceptable. Participants increased weekly the total number of steps walked. Severity of post-concussion symptoms, kinesiophobia, anxiety, and fatigue were reduced, while quality of life improved. Participants felt increased self-efficacy about physical activity
Bonn et al., 2023 (51)	Pre–post study	Session comprised circuit training, focused on common impairments in cognition, balance, endurance, vision, vestibular function, and self-management. Each session included an education section on recovery-related topics. 16 sessions at 90 min each	Canadian Occupational Performance Measure – Performance (COPM-P) Canadian Occupational Performance Measure – Satisfaction (COPM-S)	Satisfaction with self-care goals improved significantly more than leisure and productivity goals. Performance ratings were significantly greater after intervention
Bonn et al., 2025 (53)	Pre–post study	Combined physiotherapy and occupational therapy group intervention (BrainEx90); each session consisted of 40 min of circuit training, 20 min of group education and then 30 mins of additional circuit training. 16 weeks, 90 minutes once a week	COPM-P COPM-S RPQ Generalized Anxiety Disorder Scale (GAD-7)	In total, 92% experienced a clinically important improvement in COPM. Neither age, sex, days since injury, education, mechanism of injury, baseline COPM-S, GAD-7, nor RPQ scores significantly influenced satisfaction with performance of self-identified goals
Briggs et al., 2024 (63)	Pre–post study	Bottom-up treatment following the Three-component Model of Vision. The programme progression was first eye movements, accommodation, and then vergence. 8–30 weeks	Vertical/horizontal saccades, vergence jumps, near-point convergence Convergence Insufficiency Symptom Survey (CISS) COPM	Statistically significant changes were found in all outcome measurement scores after remedial vision rehabilitation, with a large effect using a *t*-test analysis
Campbell et al., 2022 (39)	Feasibility study	Four treatment areas with progression: (*I*) cervical spine, (*II*) cardiovascular, (*III*) static balance, (*IV*) dynamic balance. 6 weeks, 8 x 60-minvisits	Neurobehavioral Symptom Inventory (NSI)	Both groups reported a decrease in symptoms. The in-person group had a large effect in decreases in symptoms, whereas the tele-rehab group had a moderate effect
Connolly & Lyon, 2017 (28)	Case study	Initial therapy focused on gross vergence and feedback mechanisms and distance/near accommodative. Hart chart both at home and in the office. 18 weeks, weekly to bi-monthly therapy	Near Point Convergence (NPC) Near Point of Accommodation (NPA) Monecular Estimate Method (MEM) Negative and Positive Relate Accommodation (NRA/PRA) Smooth vergence ranges	Decreased frequency of diplopia, which did not occur daily and only at the end of the day, for 2–3 s per occurrence
Esquenazi et al., 2013 (45)	RCT	Intervention involved walking on a treadmill continuously at a prescribed speed. Every sixth minute, standardized encouragement was given. 6–8 weeks, 18 sessions 3 times/week	Overground walking, maximal velocity Stroke Impact Scale (SIS)	A statistically significant improvement in the mobility domain of the SIS was found for both groups
Fritz & Basso, 2013 (29)	Case study	Standard physiotherapy supplemented with dual-task training, motor- and cognitive-motor dual tasks. Closed environment stability and transport activities with and without intertrial variability with no manipulation. 2 weeks, 7 days in each phase	Walking While Talking Test (WWTT) Trail Making Test (TMT) Functional Independence Measure (FIM) 10-minute walk test (10MWT) BBS	WWTT: Improvement from 6 to 1 error. TMT: Improvement. FIM: Less dependent in all variables. 10MWT: 51% improvement 10 stairs up: 14% improvement 10 stairs down. BBS: Minimal change
Nguyen et al., 2014 (57)	Qualitative study	Individualized and coordinated cognitive behaviour therapy and sleep intervention, physiotherapy, and medical management. Treatment plans and participant therapy goals. 12 weeks, up to 8 sessions	Semi-structured interviews Persistent Post-Concussive Symptoms (PPCS)	Three themes describe the treatment journey: *Dissatisfaction with previous consultations*, *Perceived active ingredients of intervention*, and *Impact of interdisciplinary intervention*. Outcomes in concussion symptoms, resumption of activities and becoming more mindful, being equipped with strategies and skills to manage symptoms
Sartor-Glittenberg & Brickner, 2014 (48)	Case study	Individually tailored programme to reduce ataxia by increasing proximal stability, improve coordination of extremities, and improve balance. Interventions were modified on patient’s ability and response to treatment. 40–45 min, 3–4 days/week, decreasing to 2 days/week	BBS One Leg Stand Time (OLST) One Leg Stand Closed Eyes (SOLEC) Falls Efficacy Scale (FES) Sensory Organisation Test (NeiroCom) 6-minute walk test (6MWT) FIM	Improved to some degree on all measures and met minimal detectable change on BBS and with smallest real difference on 6MWT
Tzeng et al., 2025 (62)	RCT	Structured digital walking exercise programme, group supported by a wearable fitness tracker and personalized feedback. Exercise intensity was monitored both objectively via heart rate zones and subjectively by individual. 12 weeks	6MWT BDI-II Quality of Life (QoL) World Health Organisation Measure Quality of Life (WHOQOL-BREF)	Greater improvement in intervention group than controls on 6MWT. No significant difference on BDI and WHOQOOL-BRIEF. In the high-adherence subgroup, significant improvements were observed in functional capacity, as measured by the 6MWT, as well as in depressive symptoms, and overall quality of life
Training to restore cognitive functions (*n* = 2)				
Banerjee et al., 2021 (26)	Case study	Cognitive remediation via neurologic music therapy techniques. Use of social media for memory training. Individual and family therapy. Follow-up sessions every month. Duration not described	NIMHANS Neuropsychological Battery (NNB) BDI-II Self-evaluation	Significant improvements in neurocognitive assessments of mental speed, sustained attention, verbal fluency, verbal working memory, planning, set shifting, response inhibition, and verbal and visual learning and memory. Significant positive changes on somatic and cognitive-affective domains. Improvements in anger and mood regulation, sleep disturbance, negative recurrent thoughts, retrograde memory, generalization of functions
Palmese & Raskin, 2000 (35)	Case study	Attention retraining, use, manipulation and repetition of auditory and visual stimuli. Followed by no treatment control, sessions with an educational computer programme. 10 weeks, 1 h weekly	Consonant Trigrams (CTT) Paced Auditory Serial-Addition Test (PASAT) Symbol Digit Modalities Test (SDMT) Stroop Colour Word Interference Test (SCWT) Test of Reading Speed TORS) Digit Vigilance (DV) Revised Auditory Processing Test (R-APT)	Improvement on the CTT and the SDMT from the pre-test to the post A-test. From the post A-test to the post B-test, improvement on the PASAT. From the pre-test to the post B-test, improved performance by 1 standard deviation on the CTT and by 2 standard deviations on the CTT
Skills training to enhance cognitive functioning (*n* = 22)				
Meta cognitive skills training (*n* = 10)				
Cantor et al., 2014 (43)	RCT	STEP programme, group training in problem-solving and emotional regulation with individual sessions on attention and compensatory strategies training. 12 weeks, 9 h/week	5-step Problem Solving Inventory (SWAPS) Frontal Systems Behaviour Scale (FrSBe) Behavioural Assessment of the Dysexecutive Syndrome (BADS) Self-awareness of Deficits Interview (SAD) DERS Attention Process Training, Second Edition (APT-II) Executive function, attention, memory and affective distress, self-efficacy, social participation, quality of life	Significant treatment effects for the composite executive function measure, the FrSBe and SWAPS. No between-group differences on the neuropsychological measures or on measures of attention, emotional regulation, self-awareness, affective distress, self-efficacy, participation, or quality of life
Dawson et al., 2009 (62)	Case study	The Cognitive Orientation to Occupational Performance (CO-OP) intervention guides participants to use a meta-cognitive problem-solving strategy, the Goal-Plan-Do-Check strategy. The CO-OP emphasizes training using verbal self-instruction. Duration not described	COPM Dysexecutive Questionnaire (DEX)	Performance improved on 7 of 9 trained goals and on 4 of 7 untrained goals. Improvement was maintained at a 3-month follow-up. Participants reported significant improvement on the DEX at post-testing
Finch et al., 2017 (55)	Pilot study	Manual-based intervention designed to be goal-driven, involve group sessions with individual sessions focusing on remediating impaired social communication skills using meta-cognitive skills training. 8 weeks, 1 h/week	Profile of Pragmatic Impairment in Communication (PPIC) Goal Attainment Scale (GAS) LaTrobe Communication Questionnaire (LCQ)	PPIC: a statistically significant group change was found for 5 of the feature-summary scores across the 4 time points. GAS: 3 reached all their goals, 1 reached 2 of 3. LCQ: 2 out of 4 improved their communication skills
Fleming et al., 2005 (56)	Pre–post study	Compensatory manual-based intervention with set themes for each session, aimed at identifying potential barriers; establishing self-awareness of memory deficits; and introducing a customized compensatory tool, a cueing system and organizational strategies. 8 weeks, 1 session/week	Memory for Intentions Screening Test (MIST) Comprehensive Assessment of Prospective Memory (CAPM)) Sydney Psychosocial Reintegration Scale (SPRS) Diary entries	MIST: improvement larger than one SD for age group. CAPM: marginally improvement post. SPRS: no change. Diary entries/week: pre: 27, post: 82
Ownsworth et al., 2010 (58)	Case study	Four-session baseline of behavioural practice on meal activities. Eight sessions in the treatment phase with meta-cognitive skills training. Duration not described	Observation on error behaviour Patient Competency Rating Scale (PCRS)	Therapist-corrected errors: 76% reduction relative to baseline. Self-corrected errors: 13%–29% corrected relative to baseline. PCRS: increased between pre- and post-intervention
Rath et al., 2003 (34)	RCT	Intervention focused on problem-solving skills through emotional self-regulation and “clear thinking”. Each session followed a prepared agenda. 24 sessions, for 1 x 2-h session/week	Executive function Problem-solving self-appraisal Self-appraised emotional self-regulation and clear thinking Objective observer ratings of roleplay	The group showed significant improvement in problem solving, self-appraised clear thinking and emotional self-regulation, and objective observer ratings of roleplayed scenarios. Also showed significant improvement in visual memory, immediate recall and self-esteem
Toglia et al., 2010 (38)	Pre–post study	Multicontextual approach to cognitive rehabilitation treatment methods for teaching use of strategies across a range of meaningful activities to promote generalization and enhance functional performance. Duration not described	Awareness Questionnaire (AQ) Self-Regulation Skills Interview (SRSI) Behaviour Rating Inventory of Executive Function (BRIEF) Multiple Errands Test (MET) Executive Function Performance Test (EFPT)	Positive changes in self-regulatory skills and strategy use. General awareness of deficits remained unchanged. Examination of individual participants revealed important, varying patterns of change in strategy use, learning transfer, and self-awareness across intervention
Toglia et al., 2011 (49)	Case study	Each intervention session consisted of multiple-step everyday activities and included metacognitive techniques. Responses to questions focused on identification of challenges, and strategy generation was recorded within each intervention session during pre-activity and post-activity discussions. 5 week, 9 sessions, 75 min twice/week	SRIS AQ BRIEF MET EFPT Ratings of specific awareness Difficulties in strategy generation	Findings suggest improved functional performance and generalization of use of an external strategy despite an absence of changes in general self-awareness of deficits. This case describes the multicontext intervention process and provides clinical suggestions for working with individuals with TBI
Tsaousides et al., 2017 (37)	Pre–post study	Video-based group intervention. Comprehensive, skill-based intervention combining didactics, metacognitive strategies, and experiential training. Sessions 1–8 consisted of psychoeducation and sessions 9–24 focused on skill practice. Duration not described	DERS Positive Affect Negative Affect Schedule (PANAS) Satisfaction With Life Scale (SWLS) Problem Solving Inventory (PSI) Social Problem-Solving Inventory – Revised: Short Form (SPSI-R:S) DEX	Significant changes with large effect sizes were found for the DERS at the 12-week follow-up assessment. Significant and moderate changes were found on the SWLS, DEX, PSI, and subscales of the PANAS and SPSI-R:S

Özata Değerli et al., 2025 (32)	Case study	A multicontext approach built on the combination of remedial and compensatory techniques that focuses on generalizing learning transfer in cognitive rehabilitation. 8 week, 16 sessions, 2 sessions/week at 45 min	Loewenstein Occupational Therapy Cognitive Assessment (LOTCA) FIM Lawton Brody Instrumental Activities of Daily Living Scale (IALD) COPM EFPT	All outcome measures showed improvements. Results showed improved cognitive functioning and occupational performance, and increased independence in daily living. The client generalized the use of the strategies in different contexts and increased their occupational performance and satisfaction

Skills training directed towards community participation (*n* = 3)				
Imhoff et al., 2017 (30)	Case study	In-simulator training sessions with driving-specific automated feedback on the driving performance issued during the simulation. 5 weeks, 11 training sessions	Driving performance Simulation duration Speed regulation Lateral positioning	Speeding duration decreased during training sessions but returned to initial duration after feedback removed for the final assessment. Time spent in an incorrect lateral position decreased
Kersey et al., 2025 (64)	Feasibility study	Intervention sessions guided by participant workbook materials, including worksheets for identifying goals and making plans to achieve them. ENGAGE-TBI (a community-based group intervention further provided educational content around identifying barriers and supports, finding and using social support, and self-advocacy. 12 sessions, 2 x 90-min sessions/week	The Pittsburgh Rehabilitation Participation Scale (PRPS) Client Satisfaction Questionnaire (CSQ) Activity Card Sort (ACS) Patient-Reported Outcomes Measurement Information System (PROMIS)	Participants demonstrated clinically meaningful improvements in all measures of participation, including the ACS, change in activities retained. Minimal change was observed in social isolation and physical health. In terms of the ENGAGE-TBI structure, participants placed high value on community outings and peer mentor involvement
Tate et al., 2020 (59)	Single case experimental design	Each session included 6 elements: progress on goals; feedback on activity and performance targets; creation of opportunities for participants to reflect on their performance and reinforce use of successful strategies; confirm activities and performance levels for the following week; work with participants to develop action plans; discuss applicable strategies to enhance goal achievement. Duration not described	GAS Nottingham Leisure Questionnaire (NLQ) Revised Lubben Social Network Scale (LSNS-R) Anxiety and Stress Scales (DASS) Self-Esteem Inventory (CSEI) SWLS QOLIBRI Social Support Survey (SSS) Community Integration Measure (CIM) World Health Organisation Disability Assessment Schedule (WHODAS-2) FrSBe SPRS	Results showed large effect size (ES), although the level was not sufficient to meet GAS criteria for a clinically meaningful standard of performance. The ES for the goal was small, and the confidence interval included zero. Generalization measures showed reliable change in leisure (NLQ), community integration (CIM), quality of life (SWLS, QOLIBRI), self-esteem (CSEI), and improved mood (DASS). Increases in stress and anxiety (DASS) at follow-ups
Skills training to build psychological resources (*n* = 9)				
Bieman-Copland & Dywan, 2000 (27)	Case study	Combined implementation of behavioural therapy techniques with the development of a supportive and collaborative therapeutic alliance. Duration not described	Brock Adaptive Functioning Questionnaire (BAFQ) Challenging and maladaptive behaviours were identified	Targeted behaviours were decreased through behavioural interventions. Marked reductions in inappropriate behaviours were achieved, along with a return to supportive community living
Brown et al., 2015 (41)	RCT	Manual-based group intervention with pre-defined content regarding different aspects of advocacy for each session. Adapted for the need of each state, but key curriculum was preserved. 1 day/month for 4 consecutive months	Advocacy Behaviour Rating Scale (ABRS)	ABRS increased significantly after intervention
Faulkner et al., 2025 (60)	Feasibility study	ACTion-mTBI is a manual-based intervention built on focused ACT with the aim of addressing mTBI-related needs and promoting recovery. Duration not specified, 5 sessions	WHODAS-2 RPQ Depression Anxiety and Stress Scale – 21 (DASS-21) Valued Living Questionnaire (VLQ) Action and Acceptance Questionnaire Reactive Avoidance (AAQ-ABI (RA) Acceptance and Action Questionnaire (AAQ-II)	Feasible to implement community-based rehab. ACTion-mTBI had a significantly greater improvement in functional disability than cognitive behavioural therapy (CBT) and a significantly greater reduction in post-concussion symptoms, anxiety, and stress. Promisingly, significant improvements were found in psychological flexibility post-treatment
Faulkner et al., 2025 (61)	Qualitative study	ACTion-mTBI is a manual-based intervention built on focused ACT with the aim of addressing mTBI-related needs and promoting recovery. Duration not specified, 5 sessions	Participants’ experiences	Improved understanding of the emotional and cognitive aspects of concussion. Feeling empowered. Timing of the intervention was important
Hecker et al., 2025 (65)	Single case experimental design	Exposure exercises to challenge thoughts and expectations regarding activities the participant avoids or performs in an adapted way. 14 weeks, 14 treatment sessions	RPQ Post-Concussion Symptoms Catastrophising Scale (PCS-CS) Fear of Mental Activity Scale (FMA) Utrecht Scale for Evaluation of Rehabilitation – Participation-restriction Scale (USER-P) QoL	Satisfaction with daily life increased, and avoidance behaviour and post-concussion symptoms experienced decreased. Participants and therapists rated the intervention protocol with an average of 8.8 out of 10

King et al., 2024 (66)	Single case experimental design	Participants and therapists set up performed behavioural experiments to test participants’ fearful predictions, activities that participants indicated were bothersome, or those they avoided. 4 weeks, 22 h of therapy in total	RPQ PCS-CS FMA Hospital Anxiety and Depression (HADS) USER-P Utrecht Coping List (UCL) Eysenck Personality Questionnaire – Revised Short Scale (EPQ-RSS) Working Alliance Inventory – Short Revised (WAI-SR)	Tau-*U*, as a measure of effect, yielded significant effects for all participants on all measures when comparing baseline and intervention phases. The pooled standardized mean difference was high for all measures; symptom experience = 0.93, satisfaction with daily functioning = 1.86, and activity avoidance = −2.05
Kreutzer et al., 2018 (46)	RCT	Structured, manual-based treatment programme designed to enhance individuals’ resilience and adjustment after TBI via educational strategies, skill-building to improve communication, problem-solving, and emotional control. 5 weeks, 7 sessions, 2-month follow-up	CD-RISC Mayo Portland Adaptability Inventory – 4 (MPAI-4) Brief Symptom Inventory – 18 (BSI-18) 13-Item Stress Test	Completers had significantly improved scores for all outcomes when comparing the follow-up measures with baseline. Only the BSI-18 achieved a difference that was deemed clinically significant

Mahajan et al., 2024 (31)	Case study	A psychotherapeutic management approach involving rapport establishment, psychoeducation, addressing and exploring emotional impact and cultural beliefs, and curating techniques tailored to cognitive limitations. Duration not specified, 35–40 min/session	Subjective Units of Distress scale (SUD) Clinical observation	SUD decrease from 90 at base to 58 at last follow-up
Raina et al., 2016 (47)	RCT	Internet-delivered intervention, manual-based programme combining education, problem-solving therapy, and home assignments to teach individuals to manage self-identified fatigue-related problems. 8 weeks, 2 sessions/week	Modified Fatigue Impact Scale (MFIS) PROMIS Fatigue Severity Scale (FSS)	Intervention manual was easy to understand and use. All participants learned the intervention and could apply the intervention steps. Improvements in all questionnaires, statistical significance not reported

## RESULTS

All in all, the 2 searches generated 10,633 references. After removing 6,205 duplicates, 4,428 titles/abstracts were screened, leaving 163 articles for full-text review, whereof 14 were from the updated search. Thirty-two articles, of which 9 were from the updated search, met the eligibility criteria, with an additional 9 identified through a manual search, resulting in 41 included articles. The study-selection process and reasons for the exclusion of studies at full text are reported in Fig. 1.

**Fig. 1:**
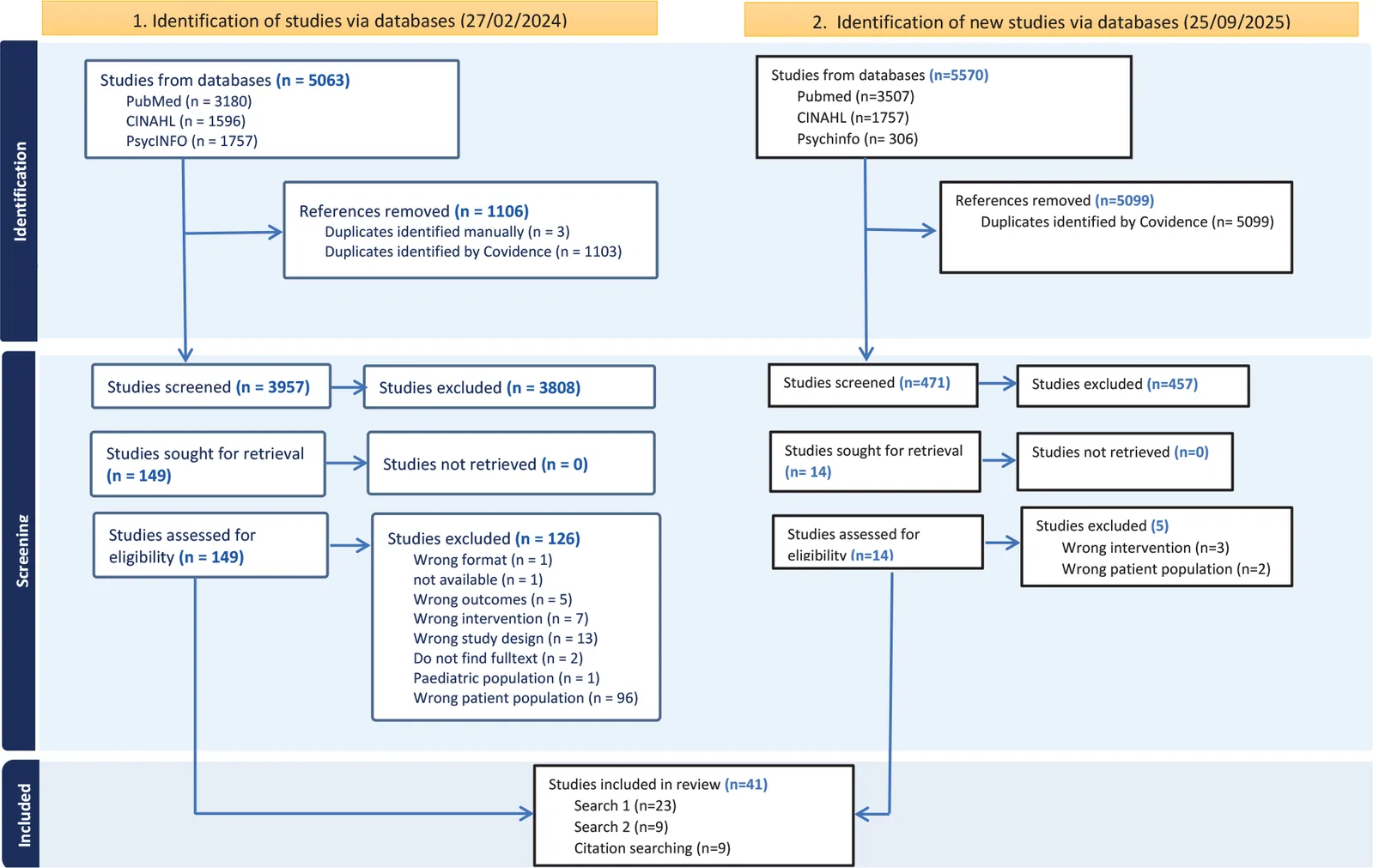
PRISMA flowchart.

None of the identified studies had a primary purpose of examining rehabilitation interventions focused on the specific needs or challenges faced by women after TBI. In several single-participant case studies, the participant was a woman (*n* = 7) (26), but the choice of a woman as the case was incidental to the primary purpose of the study. There was a trend in more recent studies to include more women and thus meet the eligibility criteria.

Studies from the United States dominated (*n* = 21) (28–49), followed by studies from Canada (*n* = 7) (27, 30, 50–54), and Australia (*n* = 5) (55–59). There were also 2 studies from India (26, 31), 2 from New Zealand (60, 61), 2 from the Netherlands (65, 66), and 1 each from Taiwan (62) and Turkey (32). The design of the studies varied; case studies were most common (*n* = 12), followed by pre–post studies (*n* = 8), RCTs (*n* = 8), feasibility studies (*n* = 4), single-case experimental designs (SCED) (*n* = 3), and mixed-method designs (*n* = 3). The studies were conducted in several service contexts, with community settings (*n* = 17) and outpatient care (*n* = 19) being the most common. The target groups for the interventions represented a variety of TBI severities. Table I provides details of the context aims, target groups within TBI, study designs, and methodologies.

The RCTs (*n* = 8) used 2 forms of control group: waitlist controls (43, 46) and active controls (34, 40, 41, 45, 47, 62). Of the RCTs using active controls, 3 studies used interventions that were considered “treatment as usual” for the target area in question (34, 41, 62), whereas 3 used study-specific control interventions (40, 45, 47). The study-specific control interventions had the same target area as the experimental intervention but differed partly in content and delivery: Esquenazi et al. (45) used a different walking support system and method of feedback; Bay et al. (40) compared their self-developed psychological lifestyle intervention focusing on stress management with a mindfulness intervention; and Raina et al. (47) compared their fatigue-directed intervention with controls receiving health education.

### Content and outcome of interventions

The final categorization consisted of 3 main categories: *Alternative and complementary interventions*, *Training to restore functions*, and *Skills training to enhance cognitive functioning*. These main categories and their underlying sub-categories (Fig. 2) are summarized below in narrative form. Table II provides detailed extracted data on the individual interventions, specific outcome measures, and outcomes for each study.

**Fig. 2:**
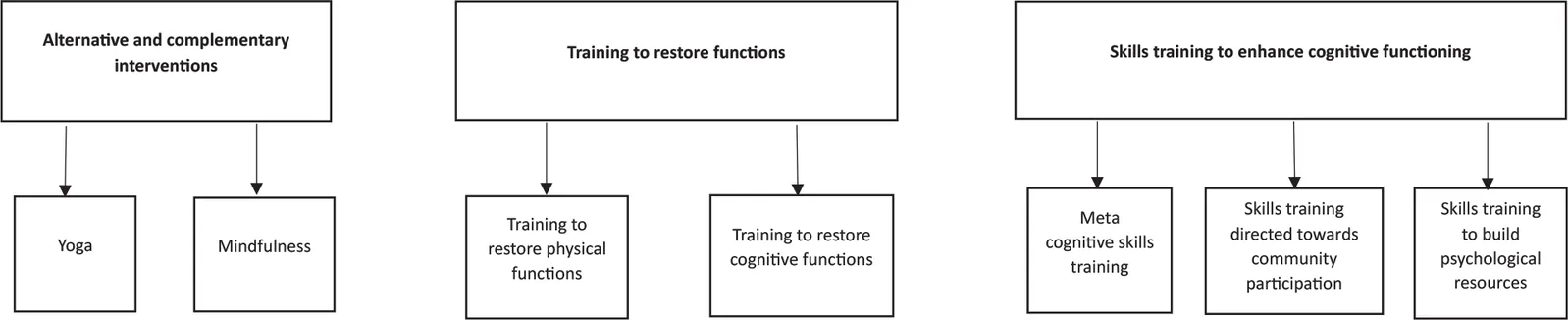
Themes and categories of the results.

### Alternative and complementary interventions

Within this category, the sub-categories of *yoga* (33, 36, 42, 44) and *mindfulness* interventions (40, 50) were identified (see Table II). When specified, intervention providers were identified as yoga therapists, with a mix of one-to-one interventions (33, 36, 40) and group interventions (42, 44, 50). All interventions had a manual or predefined structure. The duration of both intervention types varied between 6 and 12 weeks, with 1 to 2 sessions each week.

*Yoga.* Of 4 yoga interventions, all followed a classic format with breathing exercises, yoga postures, and meditation or relaxation. Two also included a psychoeducational component. Of these 2, 1 was a group-based online intervention directed not only to TBI survivors but also to caregivers and clinicians. Among other topics, the psychoeducation included education on the brain, resilience, positive thinking, and gratitude (42). The other intervention for TBI participants had a psychoeducational component aimed at cultivating resilience skills (44).

The outcome measures covered a wide range of physical, behavioural, and cognitive functions, such as physical (33) capabilities, emotional regulation (36, 42, 44), and resilience (42). Different quality of life questionnaires were also common (42, 44). Three papers used qualitative interview data alongside quantitative measures (33, 36, 44).

*Mindfulness.* Of the 2 mindfulness interventions, 1 followed a classic mindfulness structure (40), while the other followed Kabat-Zinn’s mindfulness-based stress-reduction programme in conjunction with Kolb’s experiential learning cycle, which includes group discussions (50). Outcome measures evaluated depression (40, 50), stress (50), and quality of life (50).

Overall, the results from the yoga and mindfulness interventions suggested positive outcomes on women’s quality of life, with 2 studies reporting statistically significant improvements (42, 44). Positive outcomes in physical functions were also reported, whereas the results on improvement in psychological wellbeing were inconsistent. Interview data described positive outcomes from yoga interventions regarding women’s physical functions, cognitive functions and motivation to participate in activities in the community.

### Training to restore functions

This category has two sub-categories: training to restore physical functions and training to restore cognitive functions.

*Training to restore physical functions.* Eleven papers focused on the restoration of physical functions (28, 29, 39, 45, 48, 51, 53, 54, 57, 62, 63), as outlined in Table II. Two concerned the restoration of visual convergence (28, 63), whereas 9 described interventions to restore various aspects of physical functions important for mobility, such as balance, strength, and coordination (29, 39, 45, 48, 57); cardiovascular capacity (39, 57); walking ability (45, 54, 62); and motor dual-tasking (29). The interventions were mostly provided by physiotherapists. One paper described a multidisciplinary intervention provided by a neuropsychologist, a physiotherapist, and a physician, with a focus on returning to physical activity after concussion (57).

Both interventions focusing on visual convergence (28, 63) were progressive. Outcome measures assessed convergence and accommodation. One study showed decreased diplopia (28), and the other showed statistically significant changes in all measures of vergence (63).

All interventions targeting various aspects of mobility had a progressive approach, with increasingly challenging exercises. The exercises used included cervical spine, cardiovascular, static balance, dynamic balance (39), ocular and vestibular rehabilitation with cervical management, somatosensory motor control and neck strengthening (57), treadmill walking with a bodyweight support system or a robotic gait orthosis (45), rock climbing (48), structured walking (54, 62), and transport activities in closed and open environments (29). Four interventions were delivered individually, whereas the rock-climbing intervention involved both individual and group formats (48). A circuit-training programme (51, 53) addressed common impairments in cognition, balance, endurance, vision, vestibular function, and self-management. Sessions also included education on recovery-related topics (51, 53).

Four studies (29, 45, 48, 62) evaluated balance and/or gait/walking speed, along with measures of daily functioning or independence. Overall, the results showed improvements in balance and gait/walking speed: large effect sizes regarding symptom reduction were reported by Campbell et al. (39), and statistically significant improvement in mobility was reported by Esquinazi et al. (45). Two studies evaluating self-rated goal achievement in occupational performance and satisfaction showed clinically important improvements (51, 53). In the multidisciplinary intervention (57), participants described their experiences of the treatment journey after concussion, including perceived active ingredients and the impact of the multidisciplinary approach.

*Training to restore cognitive functions.* Two case studies reported on interventions aimed at restoring attention (35) and memory (26). Both interventions used a typical restorative approach involving a hierarchical structure with progressively harder exercises with many repetitions, which were delivered individually (26, 35). The study focusing on attention used manipulation and repetition of auditory and visual stimuli (35), whereas the study focusing on memory used paper-and-pencil exercises and various music exercises, among others (26). Both case studies utilized neuropsychological tests as outcome measures (26, 35). In the work by Banerjee et al. (26), participants also self-evaluated depression, anger, and mood regulation; sleep disturbance; negative recurrent thoughts; retrograde memory; generalization of functions; and perception of “self”. Both studies showed statistically significant improvements in some or all neuropsychological tests, and Banerjee et al. (26) reported improvements in most of the participants’ self-evaluations.

### Skills training to enhance cognitive functioning

Twenty-two papers described a variety of interventions aimed at enhancing cognitive functioning (27, 30–32, 34, 37, 38, 41, 43, 46, 47, 49, 52, 55, 56, 58–61, 64–66), as shown in Table I. The papers generally stated a target area for the interventions but were often broad in approach, and some encompassed additional elements. One intervention was person-centred, with the client defining and prioritizing goal activities and the skills training tailored to manage those activities (59). The interventions were categorized into sub-categories by main target area, as follows: interventions focusing on *metacognitive skills training* (*n*=9); *skills training directed towards community participation* (*n*=3); and *skills training to build psychological resources* (*n*=10).

All papers described a clear structure for the intervention, either in the form of a manual or through defined stages. Most were delivered face-to-face in individual (27, 30, 31, 38, 49, 52, 56, 58–61, 69, 66) or group formats (38, 37, 41, 43, 46, 64) or using a combination of both (55). A few were delivered online or via video (37, 47). Among studies that specified the professionals delivering the intervention, the following were listed: psychologist/neuropsychologist (37, 43, 46, 65, 66), occupational therapists (32, 38, 47, 49, 52, 64), and speech therapist (55).

Below follows a description of the interventions by target areas.

*Metacognitive skills training.* Ten interventions focused on metacognitive skills training (32, 34, 37, 38, 43, 49, 52, 55, 56, 58), as indicated in Table II, whether in a general sense (32, 38, 49, 52), directed to a specific activity (55, 58) or directed to 1 or more predefined executive functions (43, 56). Four studies employed a broad general approach, with 3 using a multi-context approach (6) focused on the generalization of metacognitive strategies to different activities and contexts (32, 38, 49). The fourth study focused on learning a generic metacognitive strategy (CO-OP) to perform any activity (52).

One paper described an intervention aimed at improving social communication skills using metacognitive skills training and goal-based therapy in a mix of group and individual sessions (55). Three interventions used group-based metacognitive skills training and cognitive behavioural techniques to improve emotional regulation (34, 37, 43). Two included training in structured problem-solving frameworks (43, 52), and 1 also incorporated training in attention and the use of external aids (43). One study described a programme to improve prospective memory skills (56), including metacognitive skills training for developing self-awareness of memory deficits, as well as using a cueing system and organizational strategies (56).

Outcome measures varied widely between the different studies. Neuropsychological tests, questionnaires or tests regarding awareness, self-regulation, measures of goal achievement, and various measures of quality of life/well-being were commonly used. Due to the large variation in outcome measures, further information concerning the outcome measures of each study is provided in Table II. Two RCT studies showed statistically significant improvements in problem-solving, among other outcomes (34, 43). In the case studies and pre–post studies, evaluations of functional performance showed positive outcomes (32, 38, 49, 56); this also applied to the majority of the neuropsychological tests used, regardless of study design (34, 37, 43, 55, 56, 58). Outcomes regarding reintegration (34, 56), participation and quality of life (43) showed no changes.

*Skills training directed towards community participation.* Three interventions were aimed at enhancing community participation by targeting either specific activities (30, 59) or engagement in a broader sense (64). In a case study, Imhoff et al. (30) described an intervention in a car-driving simulator with automated feedback. Tate et al. (59) used a person-centred approach in a feasibility study, with the client being supported to identify and prioritize 3 meaningful activities, upon which the intervention then focused. Sessions included feedback on performance and strategies to “troubleshoot” barriers (59).

In an SCED study, Kersey et al. (64) described a manualized community-based group intervention to improve social participation. The main content involved building a participation plan with strategies for participation and identifying barriers and supports (64).

Regarding the driving simulation (30), the participants’ speeding duration decreased but returned to the initial duration after feedback was removed. Time spent in an incorrect lateral position decreased. For the intervention supporting meaningful activities (59), the results were insufficient to meet Goal-Attainment Scaling (GAS) criteria for a clinically meaningful standard of performance, but reliable changes were reported in leisure, community integration, quality of life, self-esteem, and improved mood. The intervention on social participation (64) showed clinically meaningful improvements in all measures of participation. Table II provides details of outcomes and outcome measures used.

*Skills training to build psychological resources*. Nine interventions focused on developing coping skills that targeted areas impacting life situation as a whole (27, 31, 41, 46, 47, 60, 61, 65, 66). The target areas were resilience (46), advocacy (41), psychological flexibility using acceptance and commitment therapy (ACT) (60, 61), fear avoidance (65, 66), negative behaviours related to anosognosia (27), and fatigue management (47). Many interventions were manual-based and combined treatment sessions with home assignments. Most had elements of psychoeducation and strategy use; when specified, intervention providers were identified as trained psychologists. The intervention targeting anosognosia (27) focused on behavioural therapy techniques using strategies, feedback, and reflexion in a collaborative and supporting therapeutic alliance.

The advocacy and fatigue-management interventions were group-based (41, 47). The ACT intervention (60, 61) and the fear-avoidance interventions (65, 66) were adaptations of manual-based interventions originally developed for non-TBI groups.

To evaluate outcomes, a broad range of outcome measures were used, ranging from neuropsychological tests and questionnaires to interviews on participants’ experiences. In summary, the RCT studies showed positive results. The intervention targeting resilience showed a clinically significant improvement in symptoms (46), and the intervention targeting advocacy increased advocacy behaviour significantly (41). The SCED studies on the fear-avoidance intervention showed an increase in satisfaction with daily life and a decrease in avoidance behaviours (65, 66). The case study evaluating the anosognosia interventions showed a decrease in targeted behaviours (27).

The ACT intervention was found to be feasible to implement in community-based rehabilitation, and participants’ experiences were generally positive (60, 61). The interventions targeting fatigue management were also found to be feasible for the target group (47).

## DISCUSSION

To the best of our knowledge this is the first review undertaken to identify and map evidence regarding the content and outcomes of non-pharmacological rehabilitation for women under the age of 65 with TBI. An overall and notable finding is that none of the identified studies specifically addressed women’s needs. Even though earlier research (7, 15, 16) had reported gender-based differences after TBI, no studies explicitly focused on women or aimed to examine a specific gender perspective. It was more by chance that some case studies involved a woman, as no rationale for including a woman in these studies was described. Therefore, we can assume that studies focusing on women’s specific rehabilitation needs after TBI are rare and that interventions targeting women need to be developed, studied, and trialled in rehabilitation settings for the future. However, it should be emphasized that women after TBI clearly have many of the same needs and challenges as men after TBI, and women participants were still benefiting from interventions improving cognitive efficacy, mobility, and so forth.

To address gender disparities Lian et al. (67) state that TBI rehabilitation models need to go beyond injury-related impairments and include clinical assessments “that acknowledge disrupted gender roles, responsibilities, relationships, and identities after TBI” (p. E173). In our results, most of the studies had a focus on injury-related impairments even though we could see a trend toward a more holistic view in some categories. The outcome measures used in the included studies did not reflect a gendered perspective, possibly indicating a lack of gender awareness in existing rehabilitation models.

One way to address the specific needs of women is to apply a gender lens to person-centred approaches in which the woman is involved in the whole process through goal setting, intervention, and evaluation of outcome. In this review, 1 intervention described a person-centred approach with the client defining and prioritizing goal activities that she considered meaningful and important, and the skills training was tailored to manage those activities (59). Another 4 studies (51–53, 63) used the Canadian Occupational Performance Measure (COPM) (68) for goal-setting and evaluation of outcome. The COPM is a person-centred interview-based measure and it is promising that it is used. However, it does not ensure that the intervention is person-centred and targets the person’s specific goals, nor does it ensure that the interview includes a gender perspective. In all, 5 interventions out of 41 in this study described a person-centred approach to some extent. This support research stating that person-centredness is not fully implemented in TBI rehabilitation (69, 70). Besides obstacles described in research (69, 70), a person-centred approach requires staff with knowledge of gender differences in outcomes after TBI. This is crucial to be able to ask gender-relevant questions during the goal-setting process, as the women themselves cannot be expected to spontaneously identify these. Considering the scarcity of research on understanding gender issues in women with TBI (13, 14), more knowledge is needed to help rehabilitation staff support gender-differentiated rehabilitation.

In our results, studies published in recent years included more women in their samples than previously. This may indicate an increased and more gender-specific interest in studies of rehabilitation after TBI, which is consistent with other research in this area (71). However, to improve rehabilitation for women, clinical research is needed that not only includes women but also separates and compares gender-specific patterns across the lifespan rather than just outcomes (13). Such research approaches may lead to improved support and treatment for both women and men after TBI.

Furthermore, compared with other categories in our results more studies have recently been published within the sub-category *Skills training to build psychological resources*, indicating increased interest in a relatively holistic approach to rehabilitation after TBI. Overall, the main category, *Skills training to enhance cognitive functioning*, which included the sub-category above, contained more recent studies than the categories that focused on restoration of functions. One implication may be that interventions focusing on managing difficulties in everyday life after TBI – such as through coping mechanisms and managing psychological effects after injury – are gaining increased attention in rehabilitation. Other researchers have reported a similar emphasis (72, 73). Overall, these results may indicate an increased interest in sex and gender differences and needs in rehabilitation interventions after TBI.

Given the interest in the management of psychological effects after injury, it is worth noting the lack of studies addressing interventions for women’s TBI after intimate partner violence. Earlier research has shown that women are more often affected by TBI due to intimate partner violence than men and that the numbers of affected women are undercounted (18). Such women present a complex medical picture, where traumatic experiences with conditions such as post-traumatic stress disorder (PTSD) co-vary with the effects of the TBI. Therefore, it is essential to develop and evaluate interventions to address the needs of this sub-group of women.

Earlier research on women’s experiences of living with TBI in a broader sense shows different experiences compared with men (14, 72) and one of our research questions was about women’s experiences of received rehabilitation. Among the broader group of qualitative investigations into the lived experience of women with TBI, there was little exploration of women’s experiences past treatment. According to guidelines on the evaluation of complex health interventions, studies of participant experiences should be part of evaluating interventions (74), such as those used in the area of rehabilitation, and can identify important gaps and areas for improvement. Future research studies with a focus on participant experiences are needed and can contribute to a deepened understanding of the gendered related impacts of rehabilitation interventions.

### Methodological considerations

The aim of this study was to describe interventions specifically for women; however, no such studies were found in the initial searches, and most studies had a clear bias where most participants were men. Therefore, we adjusted the eligibility criteria and accepted studies in which women comprised at least half of the sample or in which women’s results could be extracted. This approach is open to discussion, and a counterargument may be that this approach made it difficult to be conclusive about effects regarding women. Our decision was based on the assumption that, if at least half of the participants were women, then both men’s and women’s perspectives would be reflected, and the study would provide information regarding the applicability of the interventions for women.

There are several possible ways to categorize interventions, and this was a challenging task in this study, as the interventions covered a wide range of target areas and content. We chose to use a conventional approach, with a main categorization based on either restorative or compensatory content. This categorization was complicated by the fact that many interventions had elements of both. Other methods of categorization would certainly have been possible, but we consider that the chosen classification provides a useful overview.

The aim of scoping reviews is explorative and does not include a quality appraisal of included studies (22). However, it is notable that a large proportion of the included studies in this study were case studies contributing with low evidence; only 8 were RCT studies. This result highlights the need for studies with larger samples and improved designs and does also indicate that research on TBI rehabilitation in general, and regarding women specifically, needs further development.

In conclusion, the studies included in this scoping review covered a wide range of interventions from which women with TBI seem to benefit. However, the results show a lack of interventions developed for or adapted to women’s needs specifically. Further, a focus on injury-related impairments seemed to dominate the content, even though there was a trend toward a more holistic approach in recent studies. The outcome measures used did not clearly reflect a gender perspective and were mostly evaluating on impairment level rather than on activity and participation level. A possible conclusion could be that current rehabilitation models need to be developed to also include a gender perspective. To achieve this, an important clinical implication is that rehabilitation staff of all professions need to have knowledge of sex and gender differences to meet the individual needs of the person and tailor the interventions accordingly. In addition, to support and secure a gender perspective in rehabilitation, outcome measures capturing such perspectives and differences need to be developed. Further, outcomes have to a larger extent to be evaluated on activity and participation level to better capture gender perspectives.

## Supplementary Material


