# Supplementary material for: CONTENT AND OUTCOME OF NON-PHARMACOLOGICAL REHABILITATION FOR WOMEN WITH TRAUMATIC BRAIN INJURY – A SCOPING REVIEW
Source: J Rehabil Med. 2026 Oct 2;58:45767. doi: 10.2340/jrm.v58.45767 (PMC13636028; doi:10.2340/jrm.v58.45767)
Supplement: Supplementary file 1 [file JRM-58-45767-s1.pdf]

Appendix S1. Search results, Pubmed, APA Psychinfo and Ebsco Cinahl with fulltext.

Total: 5570
Duplicates identified by Covidence: 1142
Total to screen: 4428

Database: PubMed
Date searched: 2025-09-25
Limits applied: English language, Publication Date 2000-2025

Field Code	Definition
Mesh	Term from the Medline controlled vocabulary, including terms found below this term in the MeSH hierarchy
Mesh:NoExp	Does not include terms found below this term in the MeSH hierarchy
Title/Abstract	Title or abstract

Concept	#	Search	Results
Rehabilitation	1	"Activities of Daily Living"[Mesh] OR "Occupational Therapy"[Mesh] OR "Recovery of Function"[Mesh] OR "Psychosocial Intervention"[Mesh] OR "Patient Education as Topic"[Mesh]	287,962
	2	Activities of daily living[Title/Abstract] OR ADL[Title/Abstract] OR Daily living[Title/Abstract] OR Everyday activit*[Title/Abstract] OR Daily function*[Title/Abstract] OR occupational therapy[Title/Abstract] OR recovery of function*[Title/Abstract] OR function recover*[Title/Abstract] OR functional recover*[Title/Abstract] OR psycho-education*[Title/Abstract] OR psychoeducation*[Title/Abstract] OR psychosocial intervention*[Title/Abstract] OR psychological intervention*[Title/Abstract] OR patient education[Title/Abstract] OR education of patient*[Title/Abstract] OR patient teaching[Title/Abstract] OR client education[Title/Abstract]	180,381
	3	1 OR 2	404,527
Women	4	"Women"[Mesh] OR "Sex Factors"[Mesh] OR "Female"[Mesh]	10,382,446
	5	(women[Title/Abstract] OR woman[Title/Abstract] OR female*[Title/Abstract] OR sex[Title/Abstract] OR girl[Title/Abstract] OR girls[Title/Abstract] OR gender[Title/Abstract])	3,602,017
	6	4 OR 5	11,210,198
	7	"Brain Injuries"[Mesh:NoExp] OR "Head Injuries, Closed"[Mesh:NoExp] OR "Head Injuries, Penetrating"[Mesh] OR "Intracranial Hemorrhage, Traumatic"[Mesh] OR "Skull Fractures"[Mesh] OR "Brain Hemorrhage, Traumatic"[Mesh] OR "Brain Injuries, Diffuse"[Mesh] OR "Brain Injuries, Traumatic"[Mesh:NoExp] OR "Brain Injury, Chronic"[Mesh] OR "Brain Contusion"[Mesh] OR "Craniocerebral Trauma"[Mesh:NoExp]	131,633
Traumatic Brain Injury	8	(brain injur*[Title/Abstract] OR brain laceration*[Title/Abstract] OR closed head injur*[Title/Abstract] OR nonpenetrating head injur*[Title/Abstract] OR closed head trauma*[Title/Abstract] OR blunt head injur*[Title/Abstract] OR penetrating head injur*[Title/Abstract] OR penetrating head	116,194

Appendix S1. Search results, Pubmed, APA Psychinfo and Ebsco Cinahl with fulltext.

		trauma*[Title/Abstract] OR penetrating cranial trauma*[Title/Abstract] OR penetrating craniocerebral trauma*[Title/Abstract] OR penetrating brain injur*[Title/Abstract] OR traumatic intracranial hemorrhage*[Title/Abstract] OR traumatic intracranial hematoma*[Title/Abstract] OR skull fracture*[Title/Abstract] OR non-depressed skull fracture*[Title/Abstract] OR linear skull fracture*[Title/Abstract] OR traumatic brain hemorrhage*[Title/Abstract] OR traumatic cerebellar hemorrhage*[Title/Abstract] OR diffuse cerebral injur*[Title/Abstract] OR diffuse brain injur*[Title/Abstract] OR diffuse axonal brain injur*[Title/Abstract] OR traumatic brain injur*[Title/Abstract] OR brain trauma*[Title/Abstract] OR TBI[Title/Abstract] OR chronic brain injur*[Title/Abstract] OR brain contusion*[Title/Abstract] OR craniocerebral trauma*[Title/Abstract])	
	9	7 OR 8	189,371
Concepts combined	10	3 AND 6 AND 9	4,050
	11	Limit to English language, Publication Date 2000-2025	3,507

Appendix S1. Search results, Pubmed, APA Psychinfo and Ebsco Cinahl with fulltext.

Database: APA PsycInfo
Host: EBSCO
Date searched: 2025-09-25
Limits applied: English language, Publication Date 2000-2025

Field Code	Definition
DE	Subjects [exact]
XB	Title and Abstract

Concept	#	Search	Results
Rehabilitation	1	DE "Activities of Daily Living" OR DE "Occupational Therapy" OR DE "Psychosocial Interventions" OR DE "Cognitive Stimulation Therapy" OR DE "Client Education" OR DE "Recovery (Disorders)"	56,315
	2	XB (“activities of daily living” OR ADL OR “daily living” OR "everyday activit*" OR "daily function*" OR “occupational therapy” OR “recovery of function*” OR “function recover*” OR “functional recover*” OR “psycho-education*” OR psychoeducation* OR “psychosocial intervention*” OR “psychological intervention*” OR “patient education” OR “education of patient*” OR “patient teaching” OR "client education")	71,582
	3	1 OR 2	107,899
Women	4	DE "Human Females" OR DE "Battered Females" OR DE "Female Criminal Offenders" OR DE "Working Women" OR DE "Human Sex Differences" OR DE "Sex Linked Developmental Differences"	243,631
	5	XB (women OR woman OR female* OR sex OR girl OR girls OR gender)	1,010,867
	6	4 OR 5	1,023,312
Traumatic Brain Injury	7	DE "Brain Injuries" OR DE "Head Injuries" OR DE "Traumatic Brain Injury"	32,246
	8	XB ("brain injur*" OR "brain laceration*" OR "closed head injur*" OR "nonpenetrating head injur*" OR "closed head trauma*" OR "blunt head injur*" OR "penetrating head injur*" OR "penetrating head trauma*" OR "penetrating cranial trauma*" OR "penetrating craniocerebral trauma*" OR "penetrating brain injur*" OR "traumatic intracranial hemorrhage*" OR "traumatic intracranial hematoma*" OR "skull fracture*" OR "non-depressed skull fracture*" OR "linear skull fracture*" OR "traumatic brain hemorrhage*" OR "traumatic cerebellar hemorrhage*" OR "diffuse cerebral injur*" OR "diffuse brain injur*" OR "diffuse axonal brain injur*" OR "traumatic brain injur*" OR "brain trauma*" OR TBI OR "chronic brain injur*" OR "brain contusion*" OR "craniocerebral trauma*")	36,217
	9	7 OR 8	42,344
Concepts combined	10	3 AND 6 AND 9	370
	11	Limit to English language, Publication Date 2000-2025	306

Appendix S1. Search results, Pubmed, APA Psychinfo and Ebsco Cinahl with fulltext.

Database: Cinahl with Full Text
Host: EBSCO
Date searched: 2025-09-25
Limits applied: English language, Publication Date 2000-2025

Field Code	Definition
MH	Exact Subject Heading
+	Exploded Cinahl Heading
XB	Title and Abstract

Concept	#	Search	Results
Rehabilitation	1	MH "Activities of Daily Living+" OR MH "Occupational Therapy" OR MH "Recovery" OR MH "Psychosocial Intervention" OR MH "Patient Education"	225,793
	2	XB (“activities of daily living” OR ADL OR “daily living” OR “everyday activit*” OR “daily function*” OR “occupational therapy” OR “recovery of function*” OR “function recover*” OR “functional recover*” OR “psycho-education*” OR psychoeducation* OR “psychosocial Intervention*” OR “psychological intervention*” OR “patient education” OR “education of patient*” OR “patient teaching” OR “client education”)	89,650
	3	1 OR 2	276,193
Women	4	MH "Women+" OR MH "Sex Factors" OR MH "Female"	2,400,751
	5	XB (women OR woman OR female* OR sex OR girl OR girls OR gender)	1,006,271
	6	4 OR 5	2,644,681
Traumatic Brain Injury	7	MH "Brain Injuries" OR MH "Head Injuries" OR MH "Skull Fractures" OR MH "Brain Contusions"	37,322
	8	XB (“brain injur*” OR “brain laceration*” OR “closed head injur*” OR “nonpenetrating head injur*” OR “closed head trauma*” OR “blunt head injur*” OR “penetrating head injur*” OR “penetrating head trauma* ”OR “penetrating cranial trauma*” OR “penetrating craniocerebral trauma*” OR “penetrating brain injur*” OR “traumatic intracranial hemorrhage*” OR “traumatic intracranial hematoma*” OR “skull fracture*” OR “non-depressed skull fracture*” OR “linear skull fracture*” OR “traumatic brain hemorrhage*” OR “traumatic cerebellar hemorrhage*” OR “diffuse cerebral injur*” OR “diffuse brain injur*” OR “diffuse axonal brain injur*” OR “traumatic brain injur*” OR “brain trauma*” OR TBI OR “chronic brain injur*” OR “brain contusion*” OR “craniocerebral trauma*”)	35,076
	9	7 OR 8	50,429
Concepts combined	10	3 AND 6 AND 9	1,908
	11	Limit to English language, Publication Date 2000-2025	1,757
